# Genome‐wide analysis of acute traumatic spinal cord injury‐related RNA expression profiles and uncovering of a regulatory axis in spinal fibrotic scars

**DOI:** 10.1111/cpr.12951

**Published:** 2020-11-05

**Authors:** Wenzhao Wang, Jun Li, Zhengdong Zhang, Huixu Ma, Qin Li, Hai Yang, Mingxin Li, Lei Liu

**Affiliations:** ^1^ Department of Orthopedics National Clinical Research Center for Geriatrics West China Hospital Sichuan University Chengdu China

**Keywords:** acute traumatic spinal cord injury, interaction network, long non‐coding RNAs, miRNA, mRNA

## Abstract

**Objectives:**

Long non‐coding RNAs (lncRNAs) are critical for posttranscriptional and transcriptional regulation in eukaryotic cells. However, data on lncRNA expression in the lesion epicentres of spinal tissues after acute traumatic spinal cord injury (ATSCI) are scarce. We aimed to identify lncRNA expression profiles in such centres and predict latent regulatory networks.

**Materials and methods:**

High‐throughput RNA‐sequencing was used to profile the expression and regulatory patterns of lncRNAs, microRNAs and messenger RNAs (mRNAs) in an ATSCI C57BL/6 mouse model. Chromosome distributions, open reading frames (ORFs), transcript abundances, exon numbers and lengths were compared between lncRNAs and mRNAs. Gene ontology, KEGG pathways and binding networks were analysed. The findings were validated by qRT‐PCRs and luciferase assays.

**Results:**

Intronic lncRNAs were the most common differentially expressed lncRNA. Most lncRNAs had <6 exons, and lncRNAs had shorter lengths and lesser ORFs than mRNAs. MiR‐21a‐5p had the most significant differential expression and bound to the differentially expressed lncRNA ENSMUST00000195880. The microRNAs and lncRNAs with significant differential expression were screened, and a lncRNA/miRNA/mRNA interaction network was predicted, constructed and verified.

**Conclusions:**

The regulatory actions of this network may play a role in the pathophysiology of ATSCI. Our findings may lead to better understanding of potential ncRNA biomarkers and confer better therapeutic strategies for ATSCIs.

## Graphical abstract

lncENSMUST00000195880‐miR‐21a‐5p‐Smad7 was constructed as an interactive regulatory network. Overexpression of lncENSMUST00000195880 inhibited miR‐21a‐5p but promoted Smad7, thereby inhibiting activation of the TGF‐β/Smad signaling pathway.

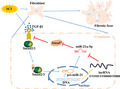

## 1 INTRODUCTION

Acute traumatic spinal cord injury (ATSCI) is considered a disabling and irreversible condition generally treated with costly and ineffective therapies.^1^ Tumour, angiopathy, infection and iatrogenic procedures can cause spinal cord injuries (SCIs), but the most common cause is trauma.^2^ SCIs can lead to a permanent loss or reduction in function below the damaged segments. In >50% of the patients who have complete SCIs, motor and sensory innervation below the damaged segments were completely lost; incompletely injured patients may retain certain neurological functions. However, there is no effective treatment for these two types of patients because of the difficulty of recovery of sensation and function.^1^, ^3^, ^4^, ^5^ Acute physical trauma is usually brief, and repair begins at the moment of damage. Neuronal, glial and angiocellular forms of epicentre necrosis occur acutely, followed by neuronal apoptosis. After 24 hours, apoptosis begins in glial cells, followed by oligodendrocytes, resulting in a non‐neuronal lesion core.^6^, ^7^, ^8^ Next, haemorrhage, ischaemia, reperfusion injury and inflammation develop. Therefore, transcription and expression of ribonucleic acid (RNA) change dramatically in the acute period.^1^ To date, scientists in the neural regeneration field have focused on dissecting out the cellular and molecular mechanisms of these processes in vivo.

Although 1%–2% of the human genome encodes proteins, most nucleotides are detectably transcribed detectably.^9^ Raquel's investigation is the first to emphasize the regulatory function of non‐coding transcripts in SCIs.^10^ Long non‐coding RNAs (lncRNAs), composed of >200 nucleotides, lack an open reading frame (ORF) and cannot be translated into protein; however, they have multiple functions, including *cis* or *trans* transcriptional regulation, nuclear domain organization and the regulation of proteins or RNA molecules.^11^ An important and major function revealed in this study was its ability to serve as competing RNA for microRNA (miRNA) binding. lncRNA has a spatio‐temporal expression pattern in embryonic stem cells and brain development; it also participates in physiological processes such as neurogenesis, cell differentiation and maturation, myelin sheath formation and synaptic plasticity.^12^ To date, few potential regulatory competitive endogenous RNA (ceRNA) networks have been revealed after SCI. One regulatory construct of note, lncRNA6032/miR‐330‐3p/Col6a1, has a potential regulatory effect in chronic SCI.^10^

The regulatory function of lncRNA has been widely acknowledged, but systemic genome‐wide analysis of ATSCI‐related lncRNA expression profiles and the precise latent regulatory networks remain unclear. Hence, this study aimed to use high‐throughput sequencing combined with a dedicated bioinformatics platform to comprehensively identify lncRNAs, miRNAs and messenger RNAs (mRNAs) expressed after ATSCI and to profile precisely the corresponding latent regulatory ceRNA network.

## 2 MATERIALS AND METHODS

### 2.1 Mouse model, sample extraction and staining

Forty‐eight clean‐grade, healthy, male C57BL/6 mice from the same litter were used for experimentation at the age of 8 weeks, each weighing 18‐22 g. They were purchased from the laboratory animal centre of Sichuan University and were randomly stratified into six groups of 8:3 groups constituted the ATSCI cohort and the other three groups constituted the Sham cohort. Eight tissue samples were harvested from each group, one of which was randomly selected for haematoxylin‐eosin (HE) staining, and the remaining were combined for RNA extraction. A standard Allen's drop SCI injury was induced as described previously.^13^ In brief, laminectomy was performed to expose the dorsal aspect of the spinal cord (T8‐T10) in SCI and Sham mice groups. Allen's drop injury (weigh of 6 g and height of 60 mm) was induced in the SCI group. The animals were sacrificed by cervical dislocation euthanasia after inducing anaesthesia with 3% pentobarbital. The spinal cord tissues at the level of the contusion injury were harvested on postoperative days 1, 3. The HE and total RNA groups were assembled 3 days post‐injury. In the HE group, as previously described previously,^13^ after being ground on ice and treatment with Trizol (Takara), the spinal cord samples were combined with lysis buffer and chloroform (Sinopharm), the middle layer centrifuged, and isopropanol (Sinopharm) added. Next, the supernatant was decanted, centrifuged with ethanol, subjected to dry precipitation and combined with RNAase‐free water (Takara). Another batch of 6 miR‐21‐Knockout (21KO) mice (Model organisms) and 6 C57BL/6 mice underwent spinal cord contusion, and 6 C57BL/6 mice underwent laminectomy without spinal cord contusion. Locomotor activity was evaluated in an open field for 10 days using the Basso Mouse Scale (BMS).^13^ Mice were sacrificed and spinal cord tissues were harvested at the lesion epicentre of the contusion injury at 10 days post‐surgery for immunohistochemical staining.

### 2.2 Construction of sequencing library and sequencing experiment

Total RNA purity was detected by a spectrophotometer (Invitrogen). Thereafter, miRNA, lncRNA, and mRNA sequencing and database construction were performed. A small RNA library was built with TruSeq Small RNA Sample Prep Kits (Illumina). Single‐end sequencing (36 or 50 bp) was performed on an Illumina Hiseq 2500 high‐throughput sequencing system (LC‐BIO). For the lncRNA and mRNA library preparation, paired‐end sequencing was performed using an Illumina Hiseq 4000 system (LC‐Bio) in the >200 bp length range.

### 2.3 Genome mapping, transcript assembly and differential expression data

Bowtie 2^14^ and Tophat 2^15^ were used to map genome reads, and StringTie^16^ was used to assemble the mapped reads of each sample and estimate the expression levels of all transcripts. miRNAs were identified in miRBase 22.1 (http://www.mirbase.org/). After identifying transcripts with coding potential by Coding Potential Calculator (CPC),^17^ Coding‐Non‐Coding‐Index (CNCI)^18^ and Pfam,^19^ the remaining transcripts with class codes i, j, o, u and x were considered to be lncRNAs. StringTie was used to determine expression levels for mRNAs and lncRNAs by calculating the fragments per kilobase of exon model per million reads mapped (FPKM). Differentially expressed miRNAs, mRNAs and lncRNAs were selected with the inclusions log_2_ (fold change) value >1, or log_2_ (fold change) value <−1 and statistically significant (*P* < .05), using the R package Ballgown.

### 2.4 Functional annotation enrichment analysis and prediction of networks

In Gene ontology (GO),^20^ the GO function compares entries in the GeneOntology (http://www.geneontology.org/) database with differential expression data. A Kyoto Encyclopedia of Genes and Genomes (KEGG) (http://www.genome.jp/kegg/)^21^ pathway was taken as the unit for pathway significance‐enrichment analysis. The lncRNAs and 3′ untranslated regions of mRNAs were predicted to be miRNA targets using TargetScan (http://www.targetscan.org/) and MiRanda (http://www.microrna.org/). Our TargetScan analysis adopted the default parameters, and the threshold set in the MiRanda analysis was Max_Energy <−10. Finally, the results of the targeted interaction analysis were taken as the points of agreement of the two algorithms. The ceRNA regulating cascades (lncRNA/miRNA/mRNA) were built by Cytoscape.

### 2.5 Cell culture and mechanical treatment

Primary spinal fibroblasts (PriCells, Wuhan, China) were cultured as previously described.^13^ Cells at passages 3‐4 were used for the subsequent experiments. After culturing to 30%–50% confluency, the cells were treated with pEXP‐RB‐Mam‐variant ENSMUST00000195880 overexpression vector and negative control controls (RiboBio) according to the manufacturer's protocol for 48 hours. Then, the cells were stimulated with TGF‐β (10 ng/mL) for 48 hours.

### 2.6 Real‐time quantitative polymerase chain reaction (qRT‐PCR)

To verify lncRNA microarray results, 2 μg of total RNA was used for cDNA synthesis with a Takara PrimeScript RT Reagent kit (Takara). A housekeeping glyceraldehyde 3‐phosphate dehydrogenase (Takara) gene was used as an endogenous control. For miRNA, The Bulge‐Loop™ miRNA reverse transcription kit (RiboBio) and a Starter kit (RiboBio) were used. Six snRNAs (RiboBio) were used as an endogenous control to quantify and normalize the results. The total cDNA was used for qRT‐PCR with SYBR Green Master Mix (Takara) on an ABI PRISM 7500 RT‐PCR System (Applied Biosystems). RNA sequences are shown in Table 1.

**TABLE 1:** Primers designed for qRT‐PCR validation. qRT‐PCR, quantitative real‐time polymerase chain reaction

Gene	Primer
ENSMUST00000195880	**F** TAGGCTGAAGAGTTGGCT
	**R** TGGAGATGGAAGTACAGTGA
mmu‐ miR‐21a‐5p	**F** CGGCGGTAGCTTATCAGACTG
	**RT**GTCGTATCCAGTGCAGGGTCCGAG GTATTCGCACTGGATACGACGTCAAC
GAPDH	**F** GTGGTGAAGCAGGCATCT
	**R** GGTGGAAGAGTGGGAGTTG
U6	**F** CTCGCTTCGGCAGCACA
	**R** AACGCTTCACGAATTTGCGT

### 2.7 Luciferase reporter assay and fluorescence in situ hybridization

293T cells were inoculated into 96‐well plates at a confluence of 70%. The plasmids pMIR‐REPORT‐ENSMUST00000195880 (WT) (LC Science) and pMIR‐REPORT‐ENSMUST00000195880 (MT1 + MT2) (LC Science) were transfected 24 hours later. Firefly and renilla duo‐luciferase reporter vector (Invitrogen) were transfected with 0.25 miRNA transfection reagent (Invitrogen) for 48 hours. A dual luciferase reporter system (E1910, Promega) was used for detection. Finally, 50 mg of pre‐mixed Stop&Glo Reagent (Invitrogen) was added and a two‐tailed *t* test was conducted after 2 seconds of reactivity. The Fluorescent in situ Hybridization Kit (RiboBio) was employed according to the manufacturer's instructions. Fluorescence was detected by the Opera Phenix HCS system (PerkinElmer).

### 2.8 Western blot analysis, immunofluorescence and immunohistochemistry

Total protein was harvested from cells and tissues as previously described,^13^ and protein concentrations were detected by BCA Protein Assay Kit (Solarbio). Proteins were subjected to sodium dodecyl sulphate polyacrylamide gel electrophoresis (Solarbio) and transferred onto polyvinylidene fluoride membranes. After blocking, the blots were incubated at 4°C overnight with the following primary antibodies: anti‐Fibronectin antibody (Abcam), type I collagen antibody (Abcam), anti‐Smad7 antibody (Cell Signaling Technology), anti‐p‐Smad2 antibody (Cell Signaling Technology), anti‐p‐Smad3 antibody (Cell Signaling Technology), anti‐Smad2/3 antibody (Cell Signaling Technology) and anti‐GAPDH antibody (1:5000; Abcam). After probing with secondary antibody (Solarbio), blots were visualized by West Pico ECL Substrate (Solarbio). Fibroblasts were stained for immunofluorescence as previously mentioned^22^; in brief, after washing, permeating and blocking, cells were incubated with Type I collagen antibody (Abcam), followed by Alexa Fluor® 594 goat anti‐rabbit IgG secondary antibody (Thermo Fisher Scientific). Immunofluorescence was analysed under a fluorescence microscope (Olympus Corporation) after staining with 4',6‐diamidino‐2‐phenylindole (DAPI; Thermo Fisher Scientific). Immunohistochemistry of spinal cord tissue sections was performed as previously described^13^; in brief, anti‐fibronectin antibody (Abcam) and anti‐Smad7 antibody (Cell Signaling Technology) were incubated on the tissue sections, subsequently incubated within secondary antibody and then stained with 3,3'‐diaminobenzidine tetrahydrochloride (Gene Tech).

### 2.9 Statistical analysis

Statistical analysis was performed using GraphPad Prism software (GraphPad Software). Data are expressed as means ± standard deviation (SD). Student's *t* test was used to assess statistical significance, and a *P*‐value <.05 was considered statistically significant.

## 3 RESULTS

### 3.1 Establishment of an Allen's spinal cord injury mouse model and RNA expression profiles

**FIGURE 1:**
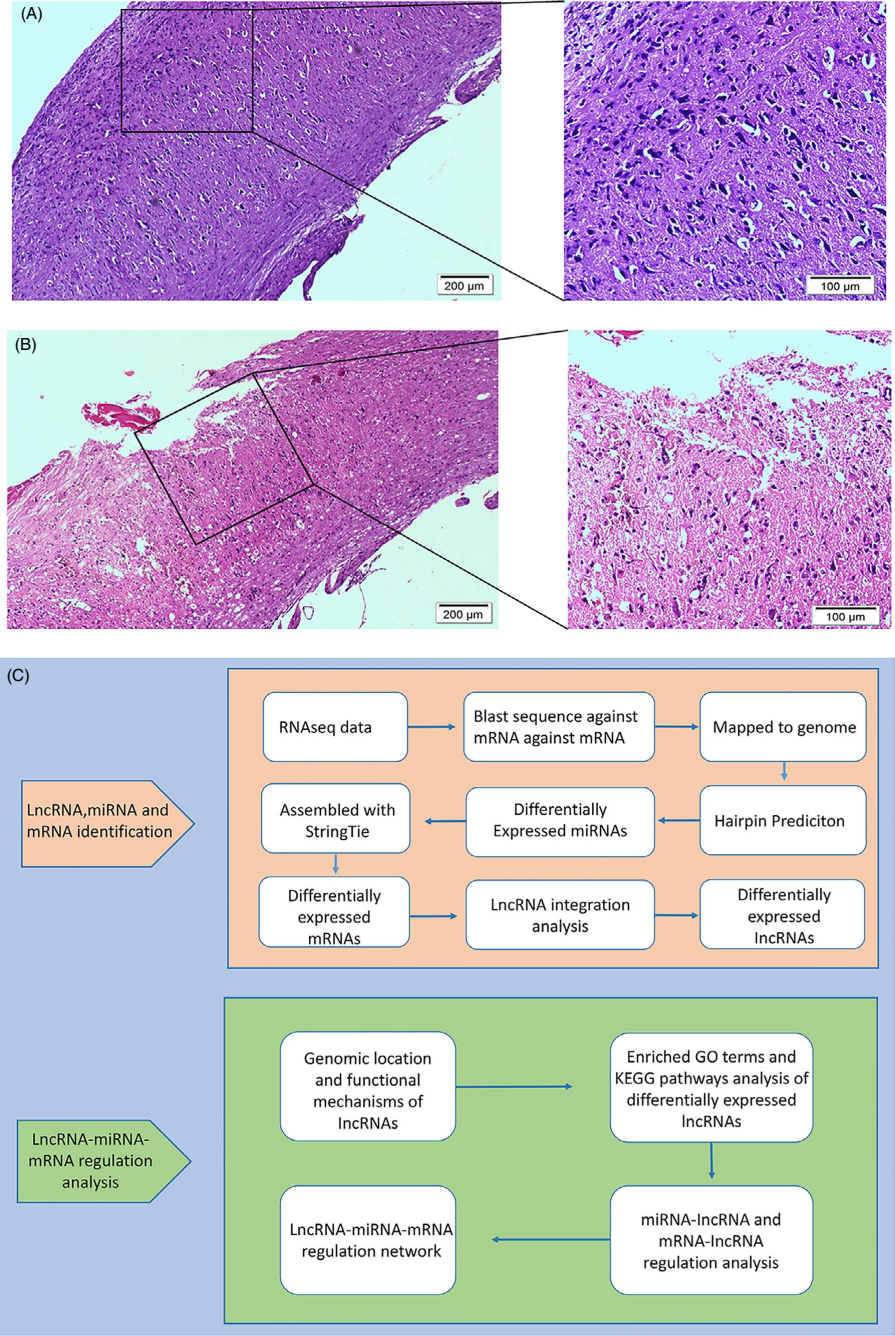
Construction of mouse SCI model and a flowchart of the identification of differently expressed RNAs HE staining of spinal cord samples. Sham group (A) and ATSCI (B) group data are shown. Overview of the analysis pipeline(C). HE, haematoxylin‐eosin; ATSCI, acute traumatic spinal cord injury; lncRNA, long noncoding RNA; mRNA, messenger RNA; miRNA, microRNA

**FIGURE 2:**
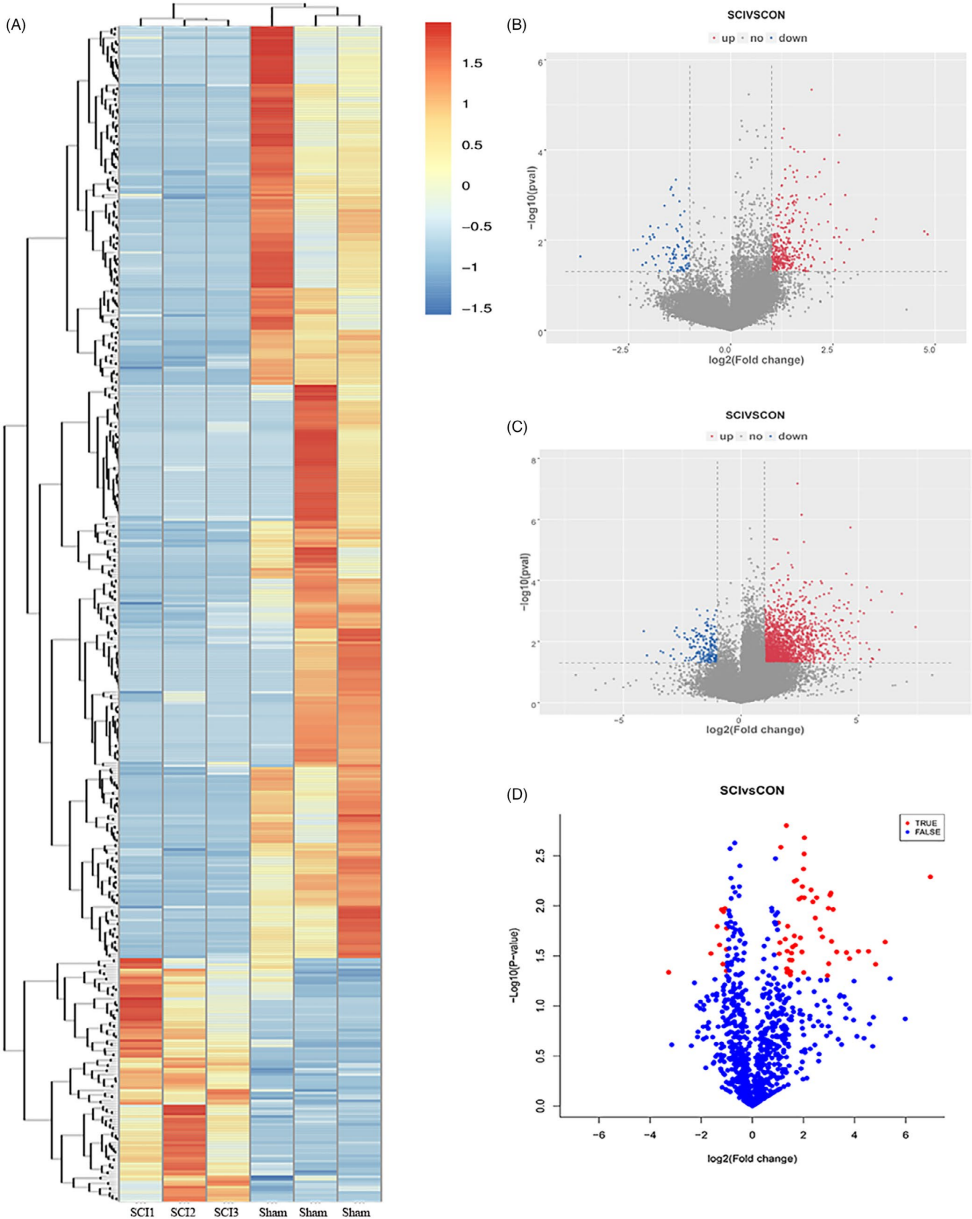
Long noncoding, micro and messenger RNA expression profiles LncRNAs are shown in the right‐hand column. Red pixels correspond to an increased abundance of the gene in the indicated sample whereas blue pixels indicate decreased levels; all differentially expressed lncRNAs show a fold change >1.5 and *P* < .05(A). Volcano plots elucidate the variance in differentially expressed lncRNA(B), mRNAs (C) and miRNAs (D) based on *P*‐values and fold changes. The x‐axis is the fold change (log 2), and the y‐axis is the *P*‐value (−log 10). Red points (fold change >2) indicate upregulated mRNAs or lncRNAs and blue points (fold change < −2) indicate downregulation in volcano plots (B, C). Red points in the volcano plot (D) indicate a fold change >2 or <−2. lncRNA, long noncoding RNA; mRNA, messenger RNA; miRNA, microRNA

Haematoxylin‐eosin ‐stained slices from the Sham group and lesion epicentres of the ATSCI group were observed under a microscope. The nuclei were stained dark blue by haematoxylin, and the cytoplasm pink and collagen fibres pale pink by eosin. In the Sham group, the spinal cord structure was clear and the spinal membrane intact without rupture, haemorrhage or signs of injury (Figure 1A). The spinal cord structure in the ATSCI group had obvious rupture indications, and the spinal membrane was damaged. Both erythrocyte dispersion and inflammatory cell infiltration caused by blood vessel rupture were visible (Figure 1B). As shown in the bioinformatics analysis pipeline workflow, lncRNAs, mRNAs and miRNAs (Figure 1C) were differentially expressed. RNA‐sequencing (RNA‐seq) data were analysed from six samples where 88‐93 million raw reads and 83‐91 million clean reads per sample were obtained. CPC, CNCI and Pfam databases were used to remove potential coding transcripts; potential lncRNAs in the samples were listed by StringTie. Differentially expressed lncRNAs are displayed as a heatmap (Figure 2A) and a volcano plot (Figure 2B). Expression profiling of mRNAs (Figure 2C) and miRNAs (Figure 2D) are shown in a volcano plot based on *P*‐values and fold changes.

### 3.2 Chromosome distributions: comparison between long non‐coding RNAs and messenger RNAs

**FIGURE 3:**
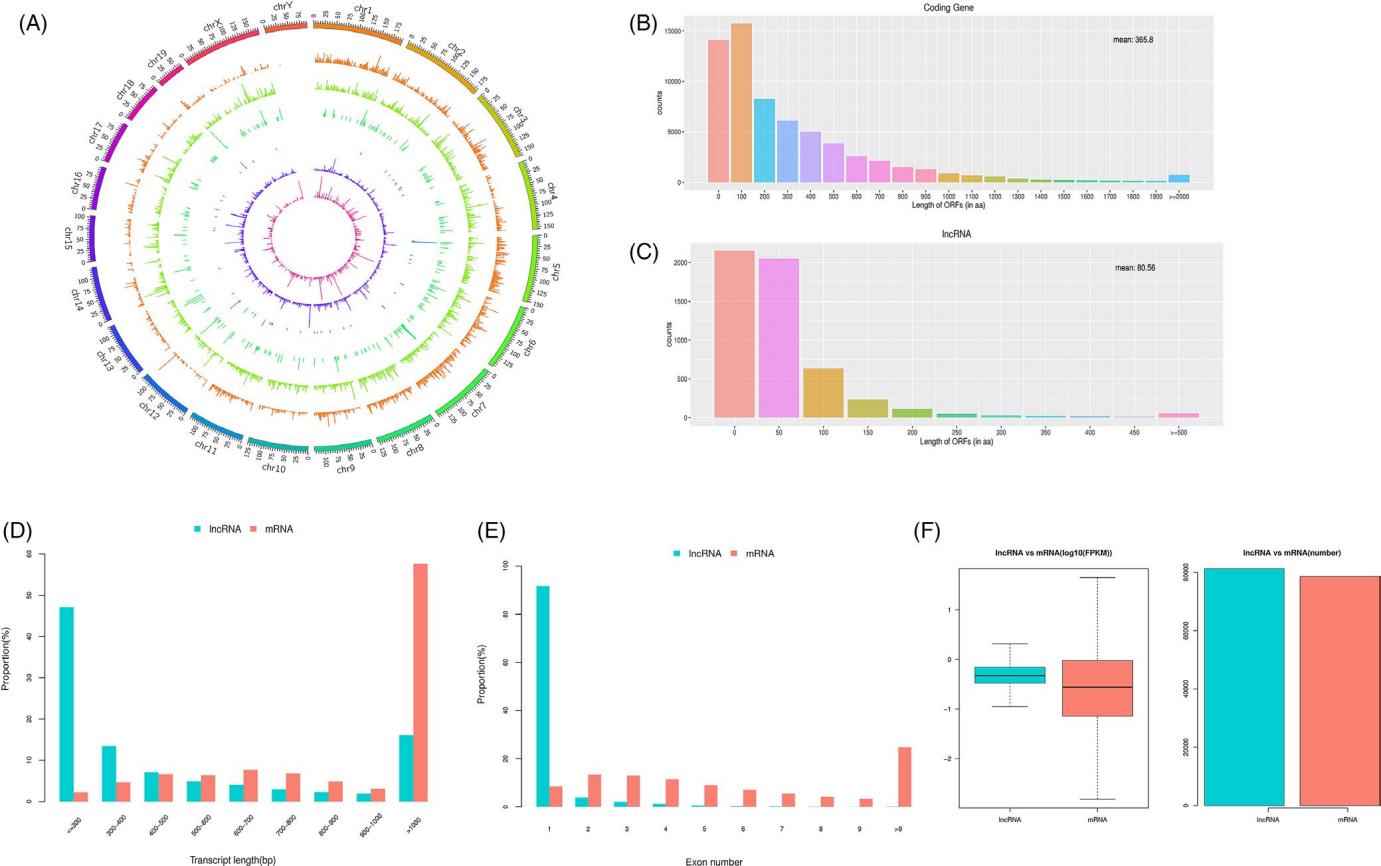
Chromosome distribution and comparison between long noncoding RNAs and messenger RNAs Distribution of 6 types of lncRNAs along each chromosome. Known lncRNAs (class code =, depicted in orange), intronic lncRNAs (class code i, depicted in light green), lncRNAs that share a reference with at least 1 splice junction (class code j, depicted in dark green), lncRNA of generic exonic overlap with a reference transcript (class code o, depicted in blue), intergenic lncRNA (class code u, depicted in violet) and antisense lncRNA (class code x, depicted in pink) are presented in physical bins of 500 kb for each chromosome (A). The ORF lengths of lncRNAs and mRNAs (B, C) are shown. The transcript length of lncRNAs and mRNAs is shown (D). Exon numbers of lncRNAs and mRNAs are shown (E). Expression levels of lncRNAs and mRNAs (F) are shown. lncRNA, long noncoding RNA; mRNA, messenger RNA

Almost all known lncRNAs were distributed over all the chromosomes, with generic exonic lncRNAs overlapping with a reference transcript (class code o) did not show significant chromosome location preferences. The percentages of the five lncRNA class codes were as follows: 71.35% intronic (a transfrag falling entirely within a reference intron), 23.2% intergenic (unknown, intergenic transcript), 4.2% antisense (exonic overlap with reference on the opposite strand), 0.97% sharing a reference with at least one splice junction, and 0.28% generic exonic with an overlap with a reference transcript (class codes i, u, x, j and o, respectively) (Figure 3A). Their properties, including ORFs, transcript abundance, exon numbers and lengths, were compared between lncRNAs and mRNAs. The lncRNAs had shorter ORFs; most mRNA ORFs were >100 nucleotides long (Figure 3B), while the ORFs of most lncRNAs were <100 nucleotides long (Figure 3C). Moreover, within length >500 bp sets, lncRNAs were shorter than mRNAs, while within length <500 bp sets, lncRNAs were longer than mRNAs (Figure 3D). Most lncRNAs had <6 exons, mRNAs had more exons and exon numbers distributed over a wider range. Moreover, numerous mRNAs had >9 exons (Figure 3E). FPKM data indicated that many lncRNAs had transcription levels similar to mRNAs (Figure 3F).

### 3.3 Enriched ontology terms and KEGG pathways of differentially expressed lncRNAs

**FIGURE 4:**
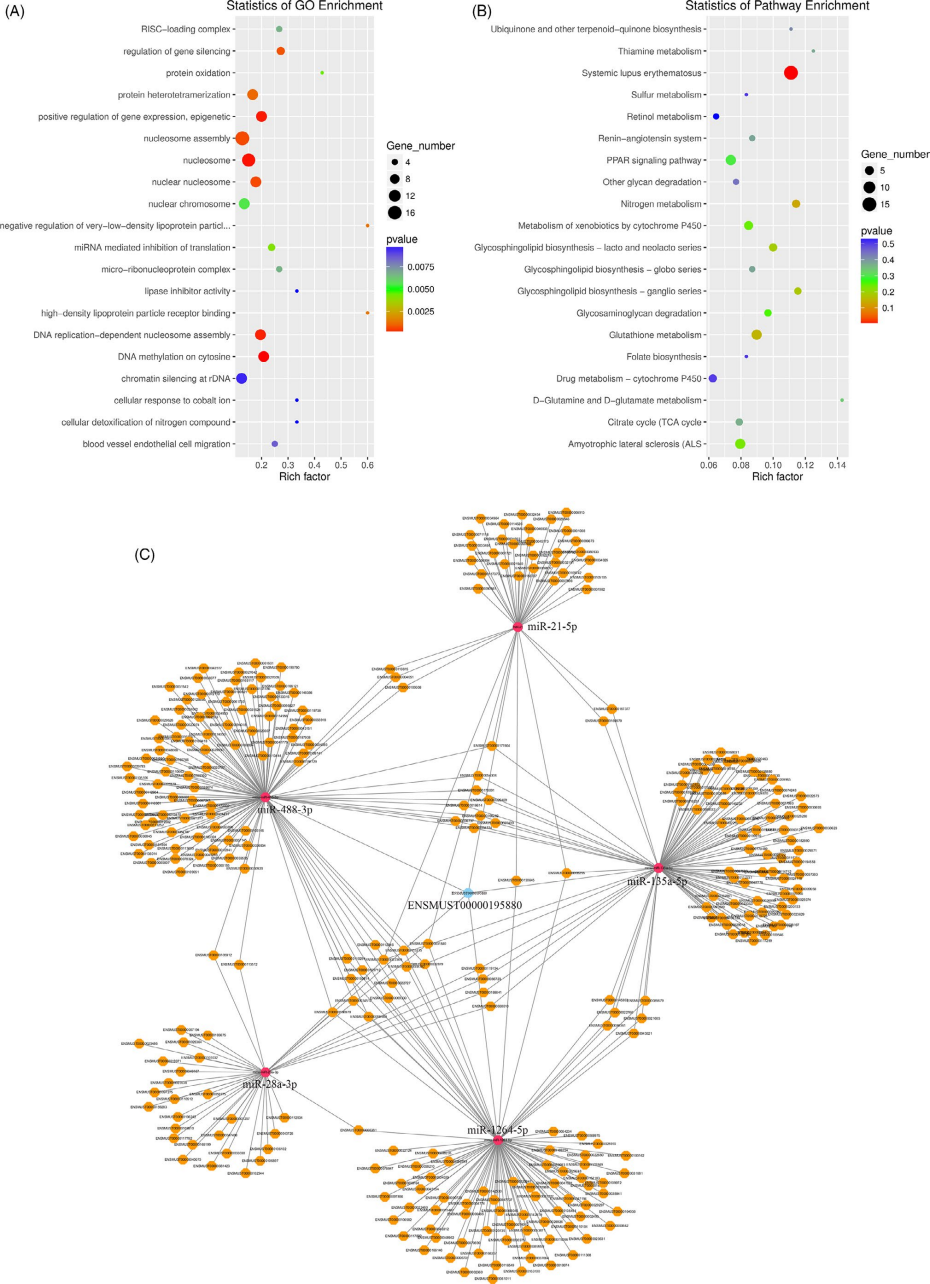
Enriched GO terms and KEGG pathways of differentially expressed lncRNAs Enriched GO terms (A) and enriched KEGG pathways (B) are shown. The rich factor is the ratio of the number of different genes to the total number of genes in the KEGG database; the higher the rich factor value, the greater the enrichment degree. GO, gene ontology; KEGG, Kyoto Encyclopedia of Genes and Genomes; lncRNA, long noncoding RNA (C) LncRNA/miRNA/mRNA regulatory interaction network of TargetScan and MiRanda prediction. lncRNA, long noncoding RNA; mRNA, messenger RNA; miRNA, microRNA

Gene ontology term enrichment can determine the main biological functions of genes via GO‐function enrichment analysis. In this study, such analysis showed that dysregulated lncRNA were associated with the following: high‐density lipoprotein particle receptor binding, negative regulation of very low‐density lipoproteins, protein oxidation, lipase inhibitor activity, cellular responses to cobalt ions and cellular detoxification of nitrogen compounds (Figure 4A). Correspondingly, dysregulated lncRNA‐associated pathways were shown by KEGG analysis to be most significantly associated with the following: D‐glutamine and D‐glutamate metabolism; thiamine metabolism; systemic lupus erythematosus; nitrogen metabolism; and glycosphingolipid biosynthesis, ganglio series (Figure 4B).

### 3.4 Prediction and construction of an lncRNA–microRNA interaction network

miR‐21a‐5p was the most highly significant expressed miRNA post‐ATSCI, with log_2_ (fold change) = 2.49 (Table 2). This is in agreement with preliminary research findings, where miR‐21a‐5p was identified as the key regulatory node of many pathophysiological processes post‐ATSCI.^5^, ^13^, ^22^ Furthermore, screened differentially expressed lncRNAs were found to target and bind to it. This targeted relationship was predicted by TargetScan (threshold 50) and MiRanda (threshold −10) software. In this study, seven differentially expressed lncRNAs were screened as follows: LncRNA ENSMUST00000195880, MSTRG.687.1, MSTRG.2861.1, MSTRG.32771.1, MSTRG.77435.1, MSTRG.106517.1 and MSTRG.127348.1 (Table 2). After a comprehensive analysis of TargetScan and MiRanda scores, lncENSMUST00000195880 was selected as a potential key lncRNA that could interact with mmu‐miR‐21a‐5p and affect the pathophysiological processed following ATSCI (Table 2). Moreover, we reverse screened several differentially expressed miRNAs that could bind with lncENSMUST00000195880: miR‐21a‐5p, miR‐28a‐3p, miR‐1246‐5p, miR‐135a‐5p and miR‐488‐3p. The interaction network of post‐ATSCI, differentially expressed lncRNAs and miRNAs was constructed with lncENSMUST00000195880 as core vertex (Figure 4C).

**TABLE 2:** The differential expression of miR‐21a‐5p and targeted lncRNAs. lncRNA, long noncoding RNA

miRNA	Regulation	log2(FC)	*P*‐value	lncRNA	Gene name	chr	Target Scanscore	Miranda Energy	Length	log2(FC)	*P*‐value	Regulation
miR‐21a‐5p	Up	2.49	.01	ENSMUST00000195880	Gm37755	chr1	90	−22.39	2179	1.1	.01	Up
miR‐21a‐5p	Up	2.49	.01	MSTRG.687.1	/	chr1	51	−12.12	1593	−1.16	.04	Down
miR‐21a‐5p	Up	2.49	.01	MSTRG.2861.1	Vwc2l	chr1	70	−12.58	759	−1.1	.02	Down
miR‐21a‐5p	Up	2.49	.01	MSTRG.32771.1	1700112E06Rik	chr14	74	−28.26	2908	1.07	.01	Up
miR‐21a‐5p	Up	2.49	.01	MSTRG.77435.1	Gm11216	chr4	54	−14.54	2563	1.59	.02	Up
miR‐21a‐5p	Up	2.49	.01	MSTRG.106517.1	Gm4593	chr7	83	−13.90	359	1.2	.04	Up
miR‐21a‐5p	Up	2.49	.01	MSTRG.127348.1	Nhs	chrX	93	−13.11	3350	−1.33	.05	Down

Abbreviations: FC, fold change, lncRNA, long noncoding RNA

### 3.5 Validation of differentially expressed ncRNA and confirmation of interaction relationships

**FIGURE 5:**
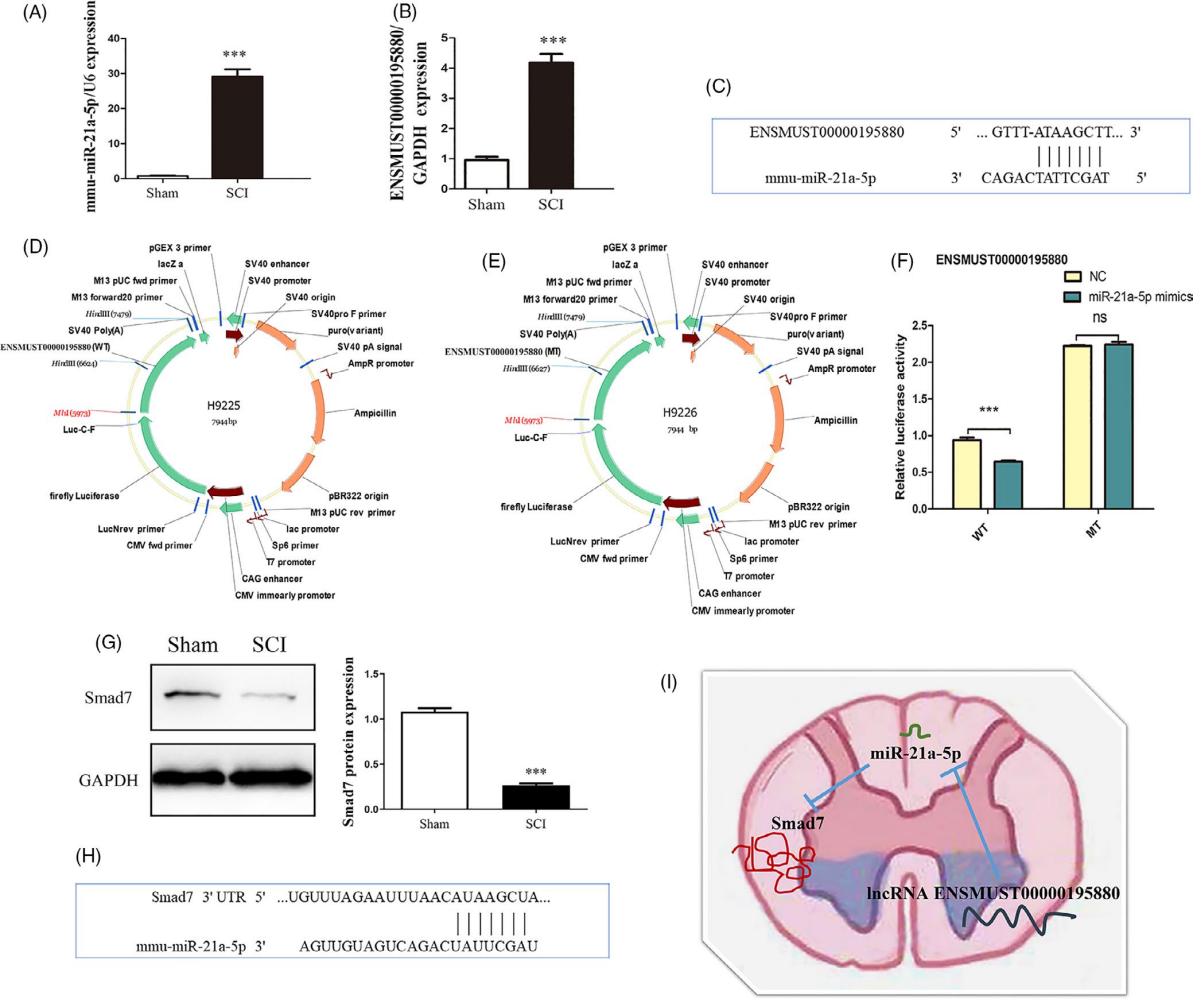
Validation of differential ncRNAs and confirmation of their relationship Expression of ENSMUST00000195880 and miR‐21a‐5p in the spinal tissues validated by qRT‐PCR (A, B). A predicted miR‐21a‐5p target site in ENSMUST00000195880 using TargetScan analysis (C) is shown. The construction of ENSMUST00000195880 (WT) and ENSMUST00000195880 (MT) luciferase plasmids (D, E) is shown. Relative luciferase expression of WT and MT ENSMUST00000195880 vectors co‐transfected with miR‐21A‐5p vectors (F) is shown. Expression of Smad7 protein in the spinal tissues validated by Western blot (G); a predicted miR‐21a‐5p target site in ENSMUST00000195880 using TargetScan analysis (H); a predicted ENSMUST00000195880/miR‐21a‐5p/Smad7 target interaction axis after SCI (I). qRT‐PCR, quantitative real‐time polymerase chain reaction, WT, wild‐type; MT, mutant; ***P < .001

To verify the accuracy of high‐throughput sequencing, we used qRT‐PCR analysis on the significant differentially expressed RNAs miR‐21a‐5p and ENSMUST00000195880. High‐throughput sequencing showed that miR‐21a‐5p and ENSMUST00000195880 were significantly upregulated after ATSCI (Table 2); qRT‐PCR results were consistent with sequencing results (Figure 5A,B). Binding was predicted between ENSMUST00000195880 and miR‐21a‐5p (Figure 5C,D). We constructed ENSMUST00000195880 (mutant; MT) and (wild‐type; WT) luciferase plasmids (Figure 5E) to verify the targeted binding relationship. We found a significant signal difference upon binding between the WT lncRNA and miR‐21a‐5p; however, the difference was abolished by the mutation, confirming that ENSMUST00000195880 could bind to miR‐21a‐5p (Figure 5F). Smad7 expression was significantly decreased after SCI (Figure 5G), and Smad7 sequence contains the binding targets of miR‐21a‐5p (Figure 5H). Prediction of targeted interaction of lncENSMUST00000195880‐miR‐21a‐5p‐smad7 in the lesion areas after SCI (Figure 5I).

### 3.6 Ethological and histological analysis of miR‐21a‐5p knockout mice

**FIGURE 6:**
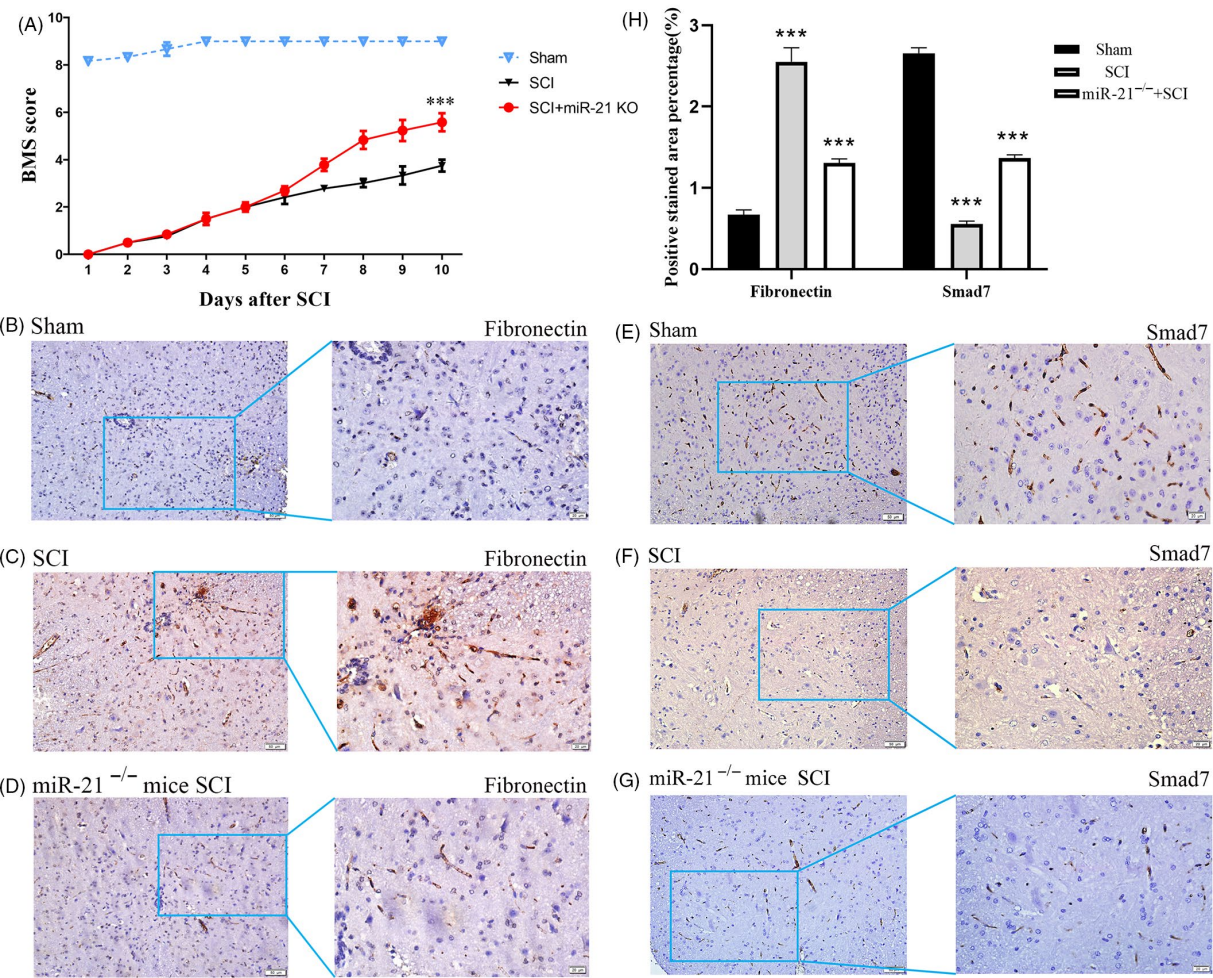
Ethological and histological analysis of miR‐21a‐5p knockout mice BMS scores indicate the motor functional index over 10 d after SCI (A). Immunohistochemistry to determine the expression of fibronectin (B, C, D) and Smad7 (E, F, G). Fibronectin and Smad7 levels in the lesion epicentre of spinal cords by immunohistochemistry (H). ***P < .001

Hindlimb motor function was assessed using the BMS from day 1 post‐surgery. The motor function of the Sham group showed an improvement on day 2 and gradually returned to normal from day 3 to day 4 post‐surgery. Five days after surgery, there was no significant difference in recovery between SCI and 21KO + SCI groups, but the 21KO + SCI group started to show a better improvement in motor function 6 days after SCI, which continued to increase over the next 3 days, gradually augmenting the distance with the SCI group (Figure 6A). Immunohistochemistry on day 10 post‐surgery showed that fibronectin was downregulated by miR‐21a‐5p inhibition, but Smad7 was simultaneously upregulated (Figure 6B‐H).

### 3.7 The validation of regulatory network in vitro

**FIGURE 7:**
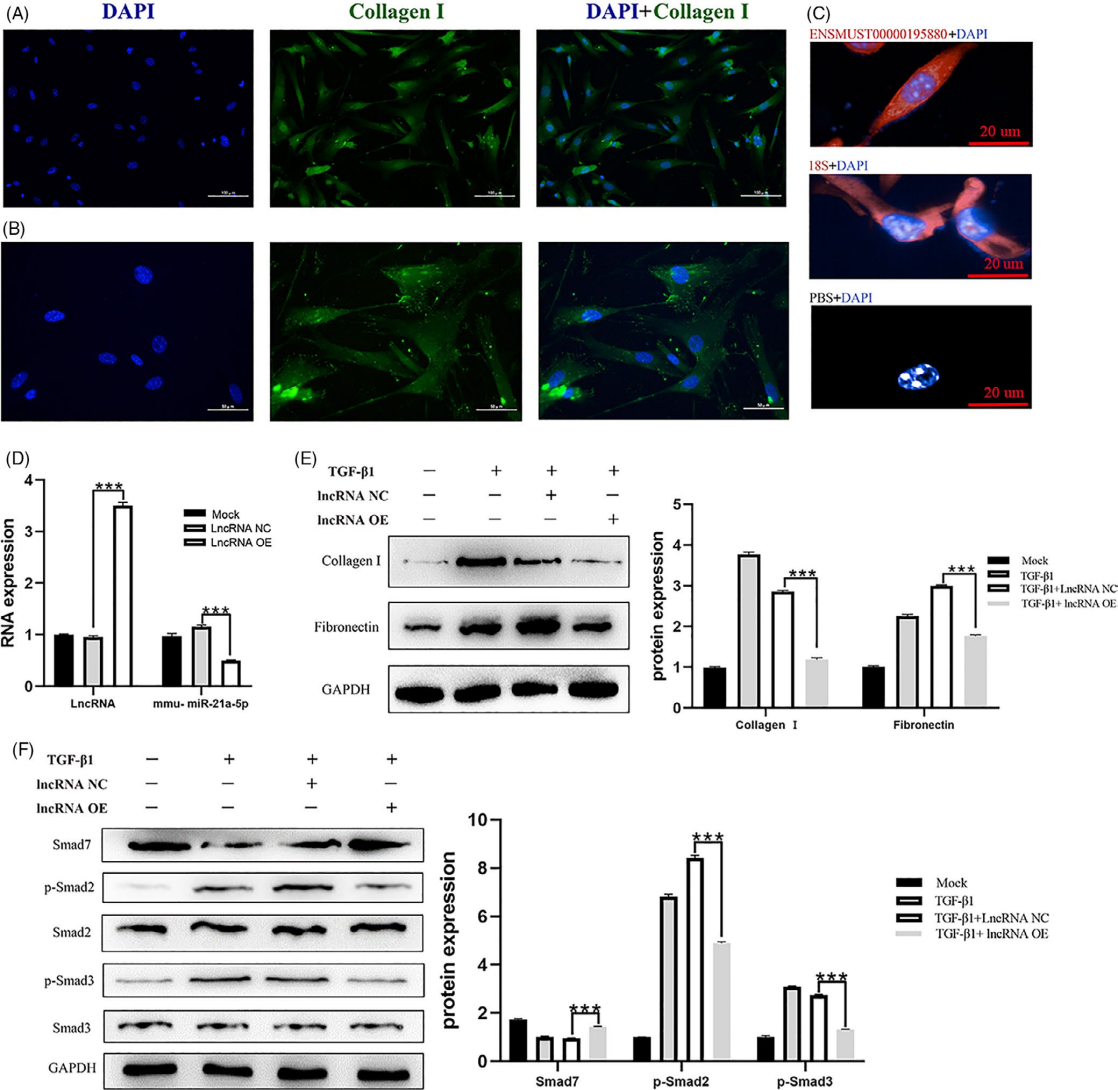
The validation of regulatory network in vitro The identification of primary spinal cord fibroblasts (A, B). the predominant localization of lncENSMUST00000195880 in primary spinal cord fibroblasts (C). Expression of miR‐21a‐5p validated by qRT‐PCR (D) and Expression of fibronectin, collagen I, Smad7 and Smad2/3 phosphorylation‐related proteins by Western blot (E, F) after lncENSMUST00000195880 overexpression. ***P < .001

**FIGURE 8:**
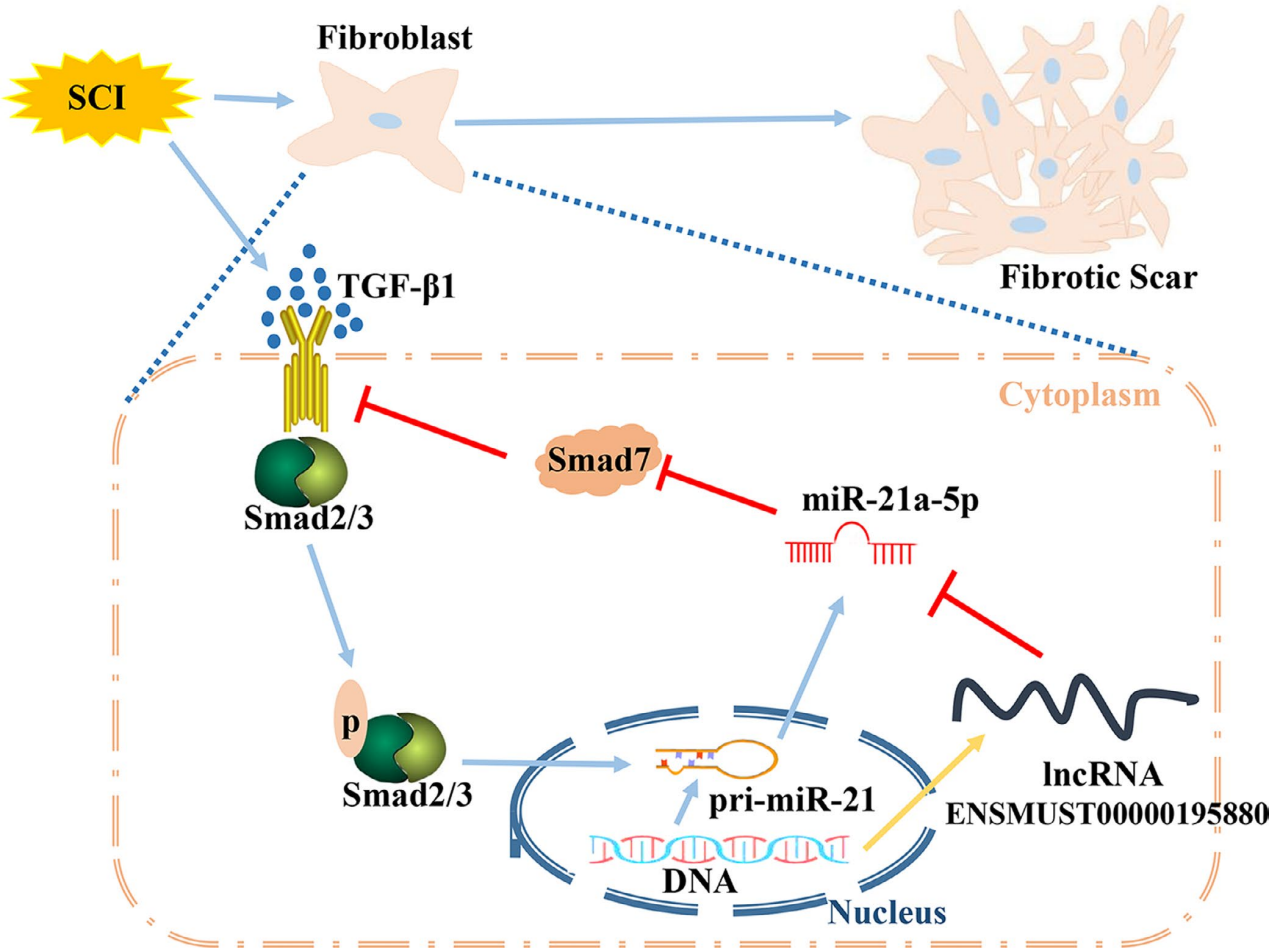
Working model of the target interaction axis after SCI After SCI, TGF‐β1 expression was increased, leading to phosphorylation of the Smad2/3 protein, thereby promoting miR‐21a‐5p upregulation. Smad7 is an inhibitor of the TGF‐β pathway that can be inhibited by miR‐21a‐5p. miR‐21a‐5p overexpression alleviates Smad7 inhibition on the TGF‐β pathway. However, the upregulated lncENSMUST00000195880, which binds with miR‐21a‐5p, suppresses this positive feedback loop

Identification of primary spinal cord fibroblasts (Figure 7A‐B), and predominant localization of lncENSMUST00000195880 in primary spinal cord fibroblasts (Figure 7C). In vitro SCI models of TGF‐β1 stimulation were constructed: the expression of miR‐21a‐5p decreased after lncENSMUST00000195880 overexpression (Figure 7D). Simultaneously, the expression of fibrosis‐related proteins fibronectin and collagen I decreased after lncRNA overexpression (Figure 7E). Overexpression of lncENSMUST00000195880 promoted Smad7 expression and inhibited Smad's pathway activation (Figure 7F). Working model of the target interaction axis after SCI (Figure 8).

## 4 DISCUSSION

This study describes lncRNA, miRNA and mRNA expression profiles, obtained via high‐throughput RNA‐seq analysis, of ATSCI spinal lesion epicentre samples from 3 ATSCI and 3 Sham groups. Despite strong scientific interest in lncRNAs, only a few studies have focused on ATSCI and lncRNAs networks. In this study, differentially expressed lncRNAs, miRNAs and mRNAs in ATSCI spinal lesion epicentres 3 days post‐SCI were identified using RNA‐seq. Sequencing data showed that ATSCI can dysregulate lncRNA, miRNA and mRNA expression. Chromosome distributions, ORFs, transcript abundance, exon numbers and lengths were compared between lncRNAs and mRNAs to study their origins. Functional GO and KEGG analyses indicated that some differentially expressed lncRNAs might play crucial regulatory roles in several mechanisms: D‐glutamine metabolism, D‐glutamate metabolism and high‐density lipoprotein particle receptor binding. A lncRNA/miRNA interaction network was predicted, constructed and preliminary edified. Thus, these results suggest that regulatory effects of altered lncRNAs and their networks might contribute to the pathophysiology of ATSCI.

ATSCIs are mainly caused by traffic accidents and falling from heights but they can also be caused by vascular lesions, tumours and iatrogenic injuries and often result in paralysis, involuntary movements, incontinence and depression.^5^ Recently, SCI patients have shown an ageing trend with the average age of injury rising from 28.7 to 37.6 years and the proportion of elderly patients rising from 4.7% to 10%.^23^ Trauma leads to the death of nerve cells and the destruction of nerve connections, thereby disrupting the excitability of upper and lower conduction nerves.^24^ The most common form of ATSCI is an immediate traumatic injury due to direct force on the spinal cord, disrupting the blood‐spinal cord barrier and leading to vasogenic spinal cord oedema, haemorrhagic transformation and disruption of axons and cell membranes.^25^, ^26^ ATSCIs include acute, subacute and chronic phases; the pathophysiology post‐SCI is biphasic and can be divided into primary and secondary phases.^27^ After the acute phase (within 3 days post‐SCI), secondary injury processes become dominant; the acute phase is likely to be the most amenable to neuroprotective interventions as it is typically the earliest point at which patients arrive at an appropriate centre to receive treatment.^28^ The delayed onset of the secondary injury phase is related to expression changes in many genes involved in vascular dysfunction, oedema, ischaemia, excitotoxicity, electrolyte shifts, free radical production, inflammation and delayed apoptotic cell death.^1^

There is increasing evidence that ncRNA plays an important role in injury progression.^25^, ^26^ Over 70% of human genes are transcribed, but <2% are translated into proteins; most of the remainder are transcribed into ncRNA.^9^ NcRNAs can be divided into two categories according to function: housekeeping ncRNAs, including small nuclear, ribosomal and transport RNA; and regulatory ncRNAs, including miRNA, circular RNA and lncRNA.^29^, ^30^, ^31^ Increasing emerging evidence indicates that an lncRNA/miRNA network has critical roles in biology and aetiology.^32^ For instance, lncRNA GAS8‐AS1 suppresses papillary thyroid carcinoma growth through the miR‐135b/CCND2 axis.^33^ Particularly in the nervous system, miRNA and lncRNA can act as regulatory or hormone‐like factors to affect communication between target cells through autocrine or paracrine pathways, thus exerting considerable influence on neurophysiology and axon regeneration.^26^, ^34^ The lncRNA SNHG5 enhances astrocyte and microglia viability by upregulating KLF4 in SCIs.^35^ A network containing XR_350851 that regulates autophagy after SCI has also been discovered.^36^

In our previous studies, we constructed in vitro TGF‐β stimulation models to explore the pathophysiological changes of spinal fibroblasts after SCI, finding the optimal duration and concentration of TGF‐β1‐induced fibroblast activation to be 10 ng/mL for 48 hours.^13^ We further demonstrated that miR‐21a‐5p acts as a positive factor for SCI recovery in the acute phase and regulates astrocyte activation of the PI3K/Akt/mTOR signalling pathway.^37^, ^38^, ^39^ Additionally, we discovered that miR‐21a‐5p could bind with Smad7, and verified that miR‐21a‐5p promotes spinal fibrosis post‐SCI via the TGF‐β/Smad signalling pathway.^22^, ^40^ Most strikingly, the astrocytes and fibroblasts in the spinal cord have been found to be key players in axon regeneration after injury.^4^, ^41^

In the present study, the lncRNAs and miRNAs differentially expressed post‐ATSCIs were determined by high‐throughput sequencing to obtain the differential expression profiles in the acute stage of injury, and the functional annotations of the related host genes were analysed. The positional relationships and correlations of expression between lncRNA and protein‐coding genes are closely related to the biological functions of lncRNA. The function of lncRNA‐related genes and the corresponding pathways in lesion epicentre tissues were identified using GO and KEGG enrichment analyses. The most significantly changed biological processes in the categories, cellular components, molecular functions and pathways, were determined. These functional predictions provide a foundation for future research into lncRNA involvement in post‐ATSCI mechanisms. miR‐21a‐5p was the most highly significant expressed miRNA post‐ATSCI, also consistent with previous research.^13^ Further, miR‐21a‐5p KO mice were used to detect the effect of miR‐21a‐5p on post‐ASCI motor function recovery. The observation period of 10 days post‐ATSCI was selected because about the subinitial stage of fibrotic scar formation takes places 10 days post‐injury.^1^, ^24^ The results showed that the motor function recovery of miR‐21a‐5p KO group was better than that of the control group. After SCI, the immunohistochemical results of the Sham group showed that fibroblasts were gradually migrating to the injury site to form a fibrous scar, but the expression of fibronectin, an important component of fibrotic scar, in the miR‐21a‐5p KO group was significantly decreased; concurrently, Smad7 expression, an inhibitor of the Smad pathway, was decreased. The above results confirm that miR‐21a‐5p plays a key role in the pathophysiological process after SCI.

Our results with the lncRNA construct lncENSMUST00000195880 were the most striking because it had the highest binding rate among all differentially expressed lncRNAs targeting miR‐21‐5p, according to predictions based on processes of the axial nervous system. Based on the one‐to‐many characteristics of binding between lncRNA and miRNA,^42^, ^43^ lncRNA/miRNA targeted interaction networks were constructed with lncENSMUST00000195880 as core vertex. Combined with our previous research,^13^ lncENSMUST00000195880/miR‐21a‐5p/Smad7 was constructed as an interactive regulatory network. lncENSMUST00000195880/miR‐21a‐5p/Smad7 axis might play an important regulatory role in the lesion epicentre after SCI. qRT‐PCR and Western blot were used to detect the differential expression of lncENSMUST00000195880 and Smad7 after SCI. Next, this interaction network was preliminarily validated in primary spinal fibroblasts by luciferase report assays, with results consistent with the prediction. Next, this interaction network was preliminarily validated in primary spinal fibroblasts, with results consistent with the prediction. Under normal conditions, TGF‐β1 expression levels in the spinal fibroblasts are relatively low. In contrast, TGF‐β1 is upregulated after SCI and specifically binds the TGF‐β1 receptors, when the downstream reaction cascade chain is activated sequentially, and the process of fibrosis gradually intensifies. The TGF‐β pathway is particularly important in fibrosis. Smad2/3 phosphorylation is activated after TGF‐β1 stimulation; although Smad7 could have inhibited this process, it was inhibited by miR‐21a‐5p overexpression. Interestingly, phosphorylated Smad2/3 can promote miR‐21a expression; therefore, a positive feedback loop is formed after SCI. However, there must be a mechanism to break this positive feedback loop and alleviate fibrosis. Most strikingly, in this study, upregulated lncENSMUST00000195880 could inhibit this positive feedback loop by binding miR‐21a‐5p after SCI. lncENSMUST00000195880 overexpression inhibited miR‐21a‐5p but promoted Smad7 expression, thereby inhibiting activation of the TGF‐β/Smad signalling pathway and fibrosis. The limitation of this study is that it only preliminarily proves the possibility of the existence of this regulatory network, but the precise regulatory relationship needs to be properly examined. In future studies, we will explore the specific biological roles, mechanisms, and signalling pathways of the lncRNA/miRNA/mRNA interaction network predicted by this study, with special focus on exploring and verifying inflammation‐related pathophysiological functions in the acute phase of ATSCI.

## CONFLICT OF INTEREST

The authors declare no conflict of interests. The sponsors had no role in the design, execution, interpretation or writing of the study, or in the decision to publish the results.

## AUTHOR CONTRIBUTIONS

Wenzhao Wang and Jun Li involved in the conceptualization; Zhengdong Zhang and Qin li performed the methodology and software; Huixu Ma performed the validation; Hai Yang analysed the data; Mingxin Li performed the data; Lei Liu involved in writing—project administration.

## ETHICAL APPROVAL

All experiments were reviewed and approved by the Ethics Committee of Sichuan University.

## Data Availability

The data that support the findings of this study are available from the corresponding author upon reasonable request.
